# The heparin-binding hemagglutinin protein of *Mycobacterium tuberculosis* is a nucleoid-associated protein

**DOI:** 10.1016/j.jbc.2023.105364

**Published:** 2023-10-19

**Authors:** Chetkar Chandra Keshavam, Saba Naz, Aanchal Gupta, Priyadarshini Sanyal, Manisha Kochar, Aakriti Gangwal, Nitika Sangwan, Nishant Kumar, Ekta Tyagi, Simran Goel, Nitesh Kumar Singh, Divya Tej Sowpati, Garima Khare, Munia Ganguli, Dominique Raze, Camille Locht, Sharmila Basu-Modak, Meetu Gupta, Vinay Kumar Nandicoori, Yogendra Singh

**Affiliations:** 1 Department of Zoology, University of Delhi, Delhi, India; 2 CSIR-Centre for Cellular and Molecular Biology, Hyderabad, India; 3 CSIR-Institute of Genomics and Integrative Biology, New Delhi, India; 4 Academy of Scientific and Innovative Research (AcSIR), CSIR- Centre for Cellular and Molecular Biology (CSIR-CCMB) Campus, Hyderabad, India; 5 Department of Biochemistry, University of Delhi South Campus, New Delhi, India; 6 Univ. Lille, CNRS, Inserm, CHU Lille, Institut Pasteur de Lille, U1019 - UMR9017 - CIIL - Centre for Infection and Immunity of Lille, Lille, France; 7 National Institute of Immunology, Aruna Asaf Ali Marg, New Delhi, India; 8 Delhi School of Public Health, Institution of Eminence, University of Delhi, Delhi, India

**Keywords:** *Mycobacterium tuberculosis*, nucleoid-associated proteins (NAPs), DNA-protein interaction, DNA damage, DNA transcription, DNA topology, genome organization

## Abstract

Nucleoid-associated proteins (NAPs) regulate multiple cellular processes such as gene expression, virulence, and dormancy throughout bacterial species. NAPs help in the survival and adaptation of *Mycobacterium tuberculosis* (*Mtb*) within the host. Fourteen NAPs have been identified in *Escherichia coli*; however, only seven NAPs are documented in *Mtb*. Given its complex lifestyle, it is reasonable to assume that *Mtb* would encode for more NAPs. Using bioinformatics tools and biochemical experiments, we have identified the heparin-binding hemagglutinin (HbhA) protein of *Mtb* as a novel sequence-independent DNA-binding protein which has previously been characterized as an adhesion molecule required for extrapulmonary dissemination. Deleting the carboxy-terminal domain of HbhA resulted in a complete loss of its DNA-binding activity. Atomic force microscopy showed HbhA-mediated architectural modulations in the DNA, which may play a regulatory role in transcription and genome organization. Our results showed that HbhA colocalizes with the nucleoid region of *Mtb*. Transcriptomics analyses of a *hbhA* KO strain revealed that it regulates the expression of ∼36% of total and ∼29% of essential genes. Deletion of *hbhA* resulted in the upregulation of ∼73% of all differentially expressed genes, belonging to multiple pathways suggesting it to be a global repressor. The results show that HbhA is a nonessential NAP regulating gene expression globally and acting as a plausible transcriptional repressor.

The linear size of bacterial genomic DNA is much larger than the bacterial cell itself. Hence, fitting the genome within a cell is a challenging task. Genetic material needs to be compacted efficiently (1000 times) (1) along with maintaining its availability to the protein machinery, regulating various cellular processes such as DNA replication, transcription, and repair. Unlike eukaryotes, these processes occur simultaneously in prokaryotes. Thus, the confined bacterial genome, known as a nucleoid, remains constantly in a dynamic state during the cell cycle (2, 3, 4). The dynamicity of the bacterial genome is maintained by histone-like proteins known as nucleoid-associated proteins (NAPs) (5, 6, 7, 8). NAPs are small, basic proteins associated with the bacterial chromosome, and their expression varies during different stages of growth (9). NAPs compact genomic DNA into microdomains as independent topological domains of ∼10 kb (10). Compaction of genomic DNA is facilitated by either bending or wrapping of DNA (HU, IHF, Fis, and Dps) or by bridging of DNA (H-NS) (5, 11). NAPs regulate multiple key cellular processes, such as transcription of genes involved in virulence and general physiology (H-NS, IHF, HU, and EspR) (6, 12, 13, 14), DNA replication (HU, IHF, and Fis) (15, 16), recombination and DNA repair (HU) (17). The number of NAPs varies among bacterial species. For example, *Escherichia coli* has at least 14 NAPs (18, 19, 20), whereas *Mtb* has only seven identified NAPs (21, 22). Considering the complex lifestyle of this pathogen, the number of identified NAPs and their described roles appear far less.

The physiological significance of NAPs in *Mtb* survival and virulence has been reported in several studies. For example, HupB is essential for the survival of *Mtb* within macrophages (23), protection of DNA from damaging agents (24), DNA repair (25), and antibiotic tolerance (26). Lsr2 is essential for survival, required for the growth of *Mtb* under normoxic, hyperoxic, and anaerobic conditions (27), protection against oxidative stress (28), isoniazid and ethambutol tolerance (29), and biofilm formation (30). EspR regulates the expression of genes involved in the typeVII secretion system that controls the secretion of many important virulence determinants of this pathogen (31), and other genes involved in survival and virulence, including genes involved in phthiocerol dimycocerosate biosynthesis (14). Another NAP, mycobacterial integration host factor (mIHF), the third most abundant protein in *Mtb*, is critical for *in vitro* growth (32, 33, 34), regulates DNA and protein synthesis, including expression of housekeeping and virulence genes (33). NapM contributes to the pathogen's survival inside macrophages during infection and in various stress conditions (35) and is involved in antibiotic tolerance (36). NapA, a recently characterized NAP from *Mtb* has been shown to regulate the expression of an operon containing genes involved in virulence (21). Based on the underrepresentation of identified NAPs and the presence of many uncharacterized genes in *Mtb*, we searched for the unidentified NAPs in *Mtb* proteome. We have identified heparin-binding hemagglutinin (HbhA) as a sequence-independent DNA-binding protein and have studied its potential role in chromatin condensation and regulation of gene expression.

## Results

### HbhA from *Mtb* is a small, basic histone-like protein

**Figure 1:**
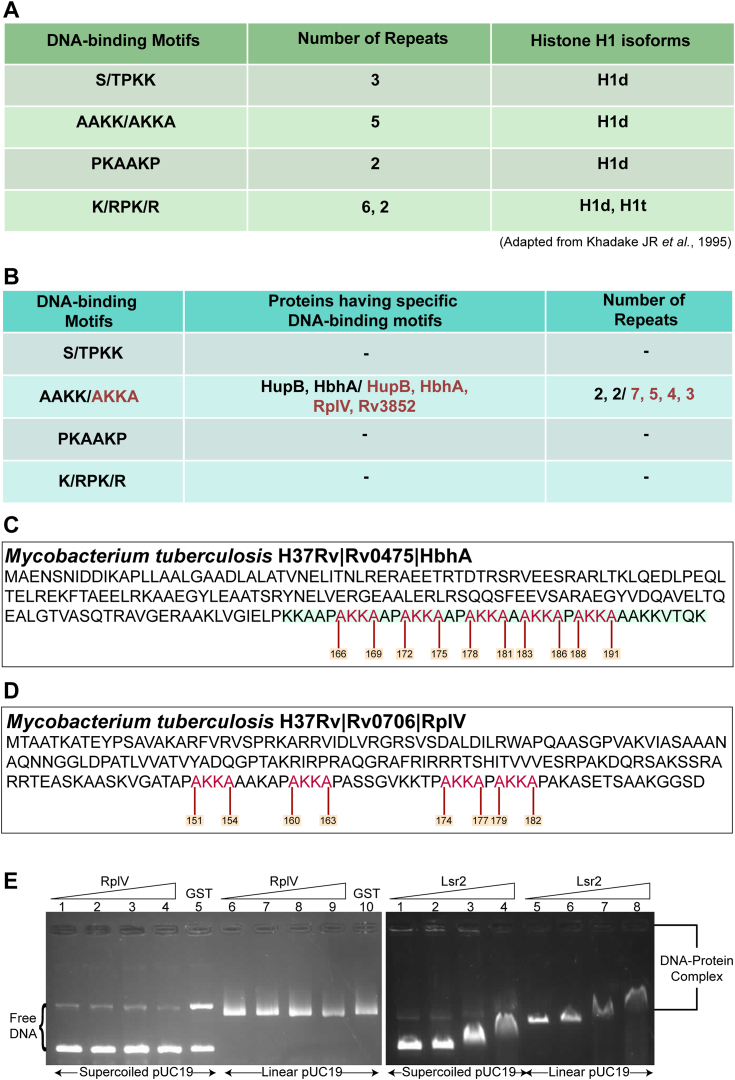
***In-silico* identification of HbhA from *Mtb* as a putative NAP.***A*, list of motifs that govern the DNA-binding ability of Histone H1 isoforms. *B*, presence of specific DNA-binding motifs in the amino acid sequence of different proteins encoded in the genome of *Mtb* and the number of repeats of DNA-binding motifs. *C* and *D*, the amino acid sequence of HbhA (*C*) and RplV (*D*) from *Mtb* shows the position of AKKA motifs. AKKA motifs are highlighted in *red*, and their positions are written in *plum*. *Green* highlighted portion of the amino acid sequence in (*C*) represents the C-terminal domain of HbhA. *E*, sequence- and structure-independent DNA-binding activity of RplV. A constant amount of supercoiled and linear forms of pUC19 DNA were incubated with increasing molar ratios (1:0, 1:50, 1:250, and 1:500) of RplV and Lsr2. Lsr2 was used as a positive control. GST at a DNA::protein molecular ratio of 1:500 was used as a negative control. GST, glutathione S-transferase; HbhA, heparin-binding hemagglutinin; *Mtb*, *Mycobacterium tuberculosis*.

**Figure S1:**
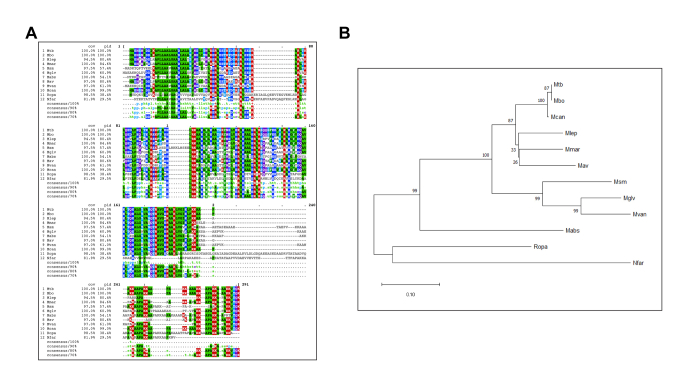
**Multiple sequence alignment (MSA) and phylogenetic analysis of HbhA from different actinobacterial species.***A*, MSA was performed using Clustal Omega (https://www.ebi.ac.uk/Tools/msa/clustalo/). Percent coverage (cov) and percent identity (pid) matrix are shown. *B*, a phylogenetic tree representing evolutionary relationships of HbhA from different actinobacterial species was generated using Neighbor-Joining analysis conducted in MEGA 11. The tree is drawn to scale with bootstrap values. Mtb, *Mycobacterium tuberculosis*; Mbo, *Mycobacterium bovis*; Mlep, *Mycobacterium leprae*; Mmar, *Mycobacterium marinum*; Msm, *Mycobacterium smegmatis*; Mglv, *Mycobacterium gilvum*; Mabs, *Mycobacterium abscessus*; Mav, *Mycobacterium avium*; Mvan, *Mycobacterium vanbaalenii*; Mcan, *Mycobacterium canetti*; Nfar, *Nocardia farcinica*; Ropa, *Rhodococcus opacus*.

Earlier studies have shown that lysine-rich repeats (Fig. 1*A*) in histone H1 isoforms are associated with their DNA-binding activity (37, 38). Therefore, to identify *Mtb* NAPs, we searched for these repeats in the *Mtb* proteome. BLAST analysis of *Mtb* proteome predicted the presence of two motifs (AAKK and AKKA) in various mycobacterial proteins. Two AAKK repeats are present in HupB and HbhA, and seven AKKA repeats are present in HupB and five in HbhA (Fig. 1*B*). The AKKA repeat motifs are also present in RplV (4 repeats), and Rv3852 (3 repeats) (Fig. 1*B*). HupB and Rv3852 have been already established as NAPs (39, 40), while the RplV protein binds specifically to 23S rRNA (https://mycobrowser.epfl.ch/genes/Rv0706). The presence of the AKKA motifs in the amino acid sequences of HbhA (Fig. 1*C*) and RplV (Fig. 1*D*) suggests that these proteins could act as NAPs. Further, the basic isoelectric point (pI-9.8 and 12.3) and small size (21.5 kDa and 20.4 kDa) of HbhA (https://mycobrowser.epfl.ch/genes/Rv0475) and RplV (https://mycobrowser.epfl.ch/genes/Rv0706) are consistent with their putative role as NAPs. We first examined the sequence-independent DNA-binding activity of RplV with supercoiled and linear forms of pUC19 DNA in an agarose gel-based mobility shift assay. The absence of a shift in the mobility pattern of pUC19 DNA in the presence of RplV (Fig. 1*E* left panel) indicated that RplV does not possess sequence-independent DNA-binding property, a hallmark feature of NAPs (6). In contrast, Lsr2, a well-characterized *Mtb* NAP (41), showed efficient binding with pUC19 DNA (Fig. 1*E* right panel). Glutathione S-transferase (GST) and Lsr2 were used as negative and positive controls, respectively. This shows that RplV does not belong to the NAPs family. Multiple sequence alignments of HbhA from various mycobacterial species and actinomycetes using CLUSTAL Omega (https://www.ebi.ac.uk/Tools/msa/clustalo/) showed that HbhA is highly conserved in actinomycetes (Fig. S1, *A* and *B*). HbhA from pathogenic mycobacterial species has been shown to act as an adhesin and is responsible for extrapulmonary dissemination of *Mtb* (42, 43).

### HbhA binds to DNA in a sequence-independent manner

**Figure 2:**
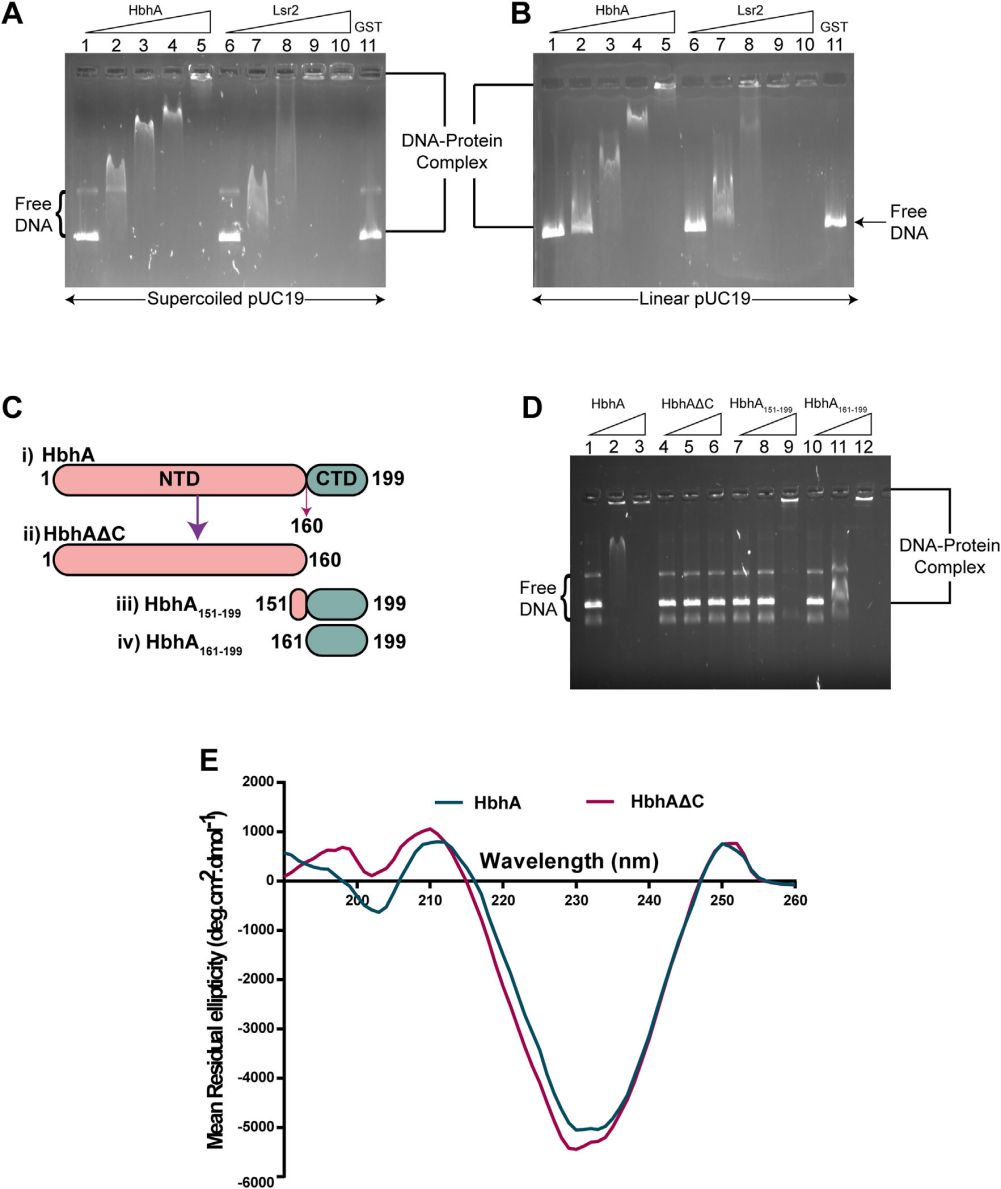
**HbhA from *Mtb* binds to DNA in a sequence- and structure-independent manner, and its C-terminal domain is DNA-binding domain.***A* and *B*, binding of HbhA with supercoiled and linear forms of pUC19 DNA, respectively. Constant amount of supercoiled and linear forms of pUC19 DNA were incubated with increasing molar ratios (1:0, 1:100, 1:250, 1:500, and 1:1000) of HbhA (lanes 1–5) and Lsr2 (lanes 6–10) with respect to pUC19. Lsr2 was used as a positive control and GST (lane 11) at a DNA::protein molar ratio of 1:1000 was used as a negative control. *C*, an illustration representing the generation of a C-terminal deleted mutant (HbhAΔC) and two N-terminal domain deleted mutants (HbhA_151-199_, and HbhA_161-199_) of HbhA. NTD and CTD represents N-terminal and C-terminal domains, respectively. *D*, constant amount of supercoiled pUC19 DNA were incubated with increasing DNA::protein molar ratios (1:0, 1:100, and 1:1000) of HbhA (lanes 1–3), HbhAΔC (lanes 4–6), HbhA_151-199_ (lanes 7–9), and HbhA_161-199_ (lanes 10–12) with respect to pUC19. Native DNA and DNA-protein complexes were resolved on 0.5% agarose gel and visualized using EtBr staining. *E*, CD spectra of HbhA and HbhAΔC. CTD, C-terminal domain; EtBr, ethidium bromide; GST, glutathione S-transferase; HbhA, heparin-binding hemagglutinin; NTD, N-terminal domain.

To evaluate the nonspecific DNA binding ability of HbhA, we performed EMSAs using pUC19 DNA as a nonspecific DNA probe. pUC19 DNA was selected due to its high AT content since NAPs bind preferentially to AT-rich DNA (6). It has been previously reported that histone-like proteins show a higher preference for binding with supercoiled DNA than to linear DNA (44, 45). Therefore, we performed EMSAs using supercoiled and linear forms of pUC19 plasmid DNA. EMSAs were performed by incubating a constant amount of DNA with varying amounts of HbhA. We observed comparable mobility shifts with supercoiled and linear pUC19 DNA with increasing amounts of HbhA (Fig. 2, *A* and *B*). We also observed that HbhA forms multiple DNA-protein complexes in a concentration-dependent manner (Fig. 2, *A* and *B*). These observations indicate a complex much larger than expected by the association of a single molecule of HbhA with plasmid DNA, suggesting the binding of multiple HbhA molecules with a single DNA molecule in a sequence-independent fashion (Fig. 2, *A* and *B*). GST and Lsr2 were used as the negative and positive controls, respectively.

### The C-terminal domain of HbhA is required for DNA binding

Earlier studies have revealed the presence of three functional domains in HbhA: a hydrophobic domain of 18 amino acid residues in the N-proximal region of the protein, an α-helical coiled-coil domain of 81 amino acid residues and a C-terminal domain (161–199 amino acid residues) which is rich in Lys-Pro-Ala residues (42, 46, 47, 48). We observed the presence of five repeats of the AKKA motif in the C-terminal domain of HbhA (Fig. 1*C*). As the presence of AKKA and several similar motifs in histones and NAPs has been linked to their DNA-binding abilities (24, 37, 38, 40, 49, 50, 51, 52), we investigated the role of AKKA motifs in the DNA-binding activity of HbhA. We, therefore, constructed a mutant with a C-terminal deletion (HbhAΔC) and two mutants with N-terminal deletions (HbhA_151-199_ and HbhA_161-199_) (Fig. 2*C*). We observed a complete loss in the mobility shifts of pUC19 DNA in the presence of HbhAΔC (Fig. 2*D*). In contrast, mobility shifts of pUC19 DNA were observed in the presence of HbhA_151-199_ and HbhA_161-199_ and were comparable to that of HbhA (Fig. 2*D*). To rule out the possibility of mutation-induced structural alterations in HbhAΔC, circular dichroism spectra were determined to examine the secondary structures of HbhA and HbhAΔC. Superimposed spectra of HbhA and HbhAΔC showed similar patterns (Fig. 2*E*). These results indicate that the C-terminal domain of HbhA containing AKKA repeat motifs is responsible for its DNA-binding activity.

### HbhA binding to DNA leads to DNA looping and bending

**Figure 3:**
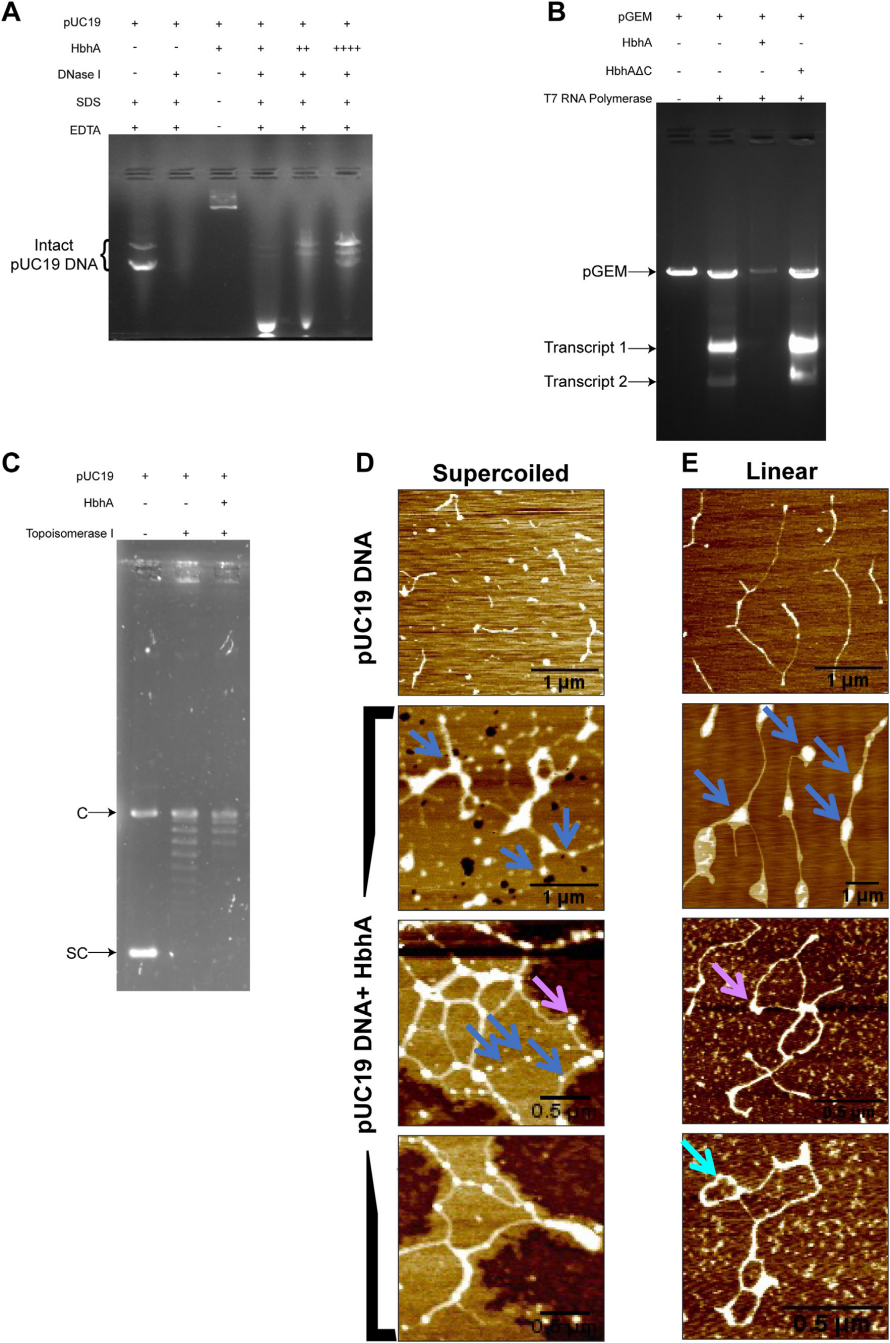
**HbhA possesses characteristic features of a NAP.***A*, agarose gel image showing HbhA mediated protection of DNA from DNaseI enzymatic activity. Increasing DNA::HbhA molecular ratios enhanced DNA protection from DNaseI activity. +, ++, and ++++ denotes 1:250, 1:500, and 1:1000 DNA::HbhA molar ratios, respectively. *B*, agarose gel image showing inhibitory effect of HbhA on *in vitro* transcription reaction mediated by T7 RNA polymerase. Expected sizes of transcripts 1 and 2 are ∼2368 nucleotides and ∼1042 nucleotides, respectively. + and - denotes the presence and absence of specific components in the reaction mixture. *C*, agarose gel image showing the effect of HbhA on topoisomerase I-mediated relaxation of pUC19 DNA. + and - denotes the presence and absence of specific components in the reaction mixture. C and SC denote the covalently closed circular and supercoiled forms of pUC19 DNA, respectively. *D* and *E*, AFM images showing HbhA-mediated architectural modulations in supercoiled and linear forms of pUC19 DNA, respectively. 1:250 DNA::HbhA molar ratio was used to visualize the architectural effect of HbhA on pUC19 DNA. *Upper panel* in (*D* and *E*) denotes supercoiled and linear forms of pUC19 DNA alone, respectively, in 1× EMSA buffer. *Blue arrows* denote beads on a string like structure, *lavender arrows* denote bending of DNA molecule, *cyan arrow* denotes looping of DNA molecules. Scale bars are denoted in respective panels. AFM, atomic force microscopy; HbhA, heparin-binding hemagglutinin; NAP, nucleoid-associated proteins.

Histones and histone-like proteins protect DNA from degradation by DNA-damaging agents such as DNaseI (24, 29, 53, 54, 55). To test whether HbhA binding to DNA results in protection from DNaseI, we incubated HbhA with 500 ng of supercoiled pUC19 DNA in an increasing DNA::protein molar ratio, followed by treatment with 1 U of DNaseI. In the absence of HbhA, treatment of pUC19 DNA with DNaseI resulted in degradation of DNA, while in its presence, increased protection was observed with increasing DNA::protein molar ratios with almost complete protection at the 1:1000 molar ratio (Fig. 3*A*). These results indicate that HbhA protects DNA from DNaseI degradation.

Histone and other NAPs are involved in the *in-vitro* transcription regulation (56, 57). Lsr2, a known nucleoid-associated protein from *Mtb*, has also been shown to inhibit *in-vitro* transcription using T7 promoter system (29). To check whether HbhA could be a transcriptional regulator, we measured the *in-vitro* transcription regulatory activities of HbhA and observed that HbhA inhibits transcription, while HbhAΔC failed to inhibit transcription (Fig. 3*B*). Histone-like proteins are known to modulate the topological features of DNA in the presence of topoisomerase (21, 29, 44, 58, 59). To determine whether HbhA can also perform such a function, we incubated pUC19 DNA with topoisomerase I to relax the supercoiled DNA and added HbhA. We observed that HbhA enhanced the relaxation activity of topoisomerase I (Fig. 3*C*).

**Figure S2:**
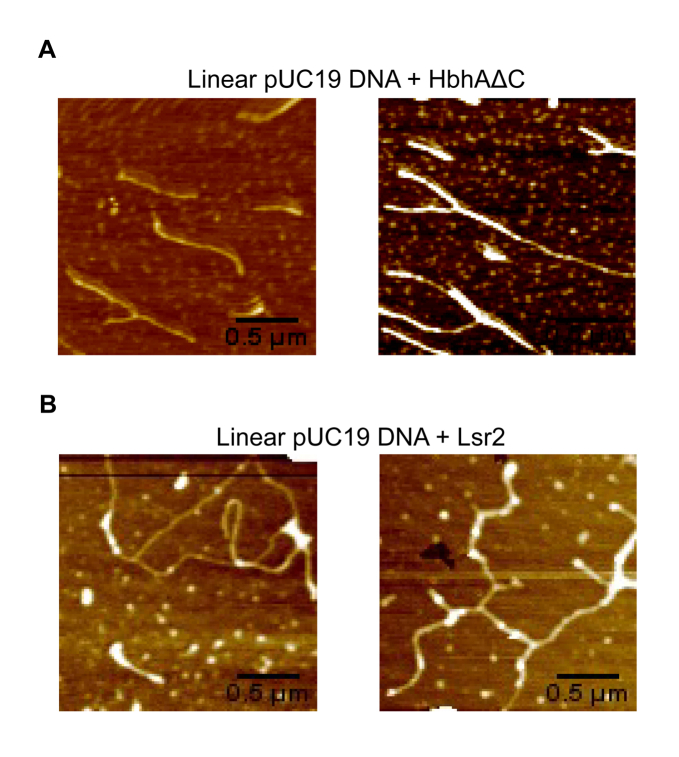
**Effect of HbhAΔC and Lsr2 on the architectural features of linear pUC19 DNA.** Effect of (*A*) HbhAΔC and (*B*) Lsr2 on the architectural features of linear pUC19 DNA. Lsr2 was used as positive control for AFM imaging. Scale bars are denoted in respective panels.

To gain further insight into any possible conformational changes in the DNA induced by the binding of HbhA, we used atomic force microscopy. We observed HbhA-induced changes in linear and supercoiled plasmid DNA morphology. At various regions, it is observed that the DNA bound to HbhA proteins appeared as beads on string structure (bright areas indicated by blue arrows in Fig. 3, *D* and *E*). In some regions, there was looping (cyan arrow) and bending (lavender arrow) of the DNA as well (Fig. 3, *D* and *E*). This is likely induced by the nonspecific binding of HbhA, since it appears to be randomly bound on the DNA filaments. Without HbhA, the DNA appears as filaments with uniform height (Fig. 3, *D* and *E*, upper panels). No conformational changes in the DNA occurred in the presence of HbhAΔC (Fig. S2*A*). Lsr2 was used as a positive control for these experiments, showing that Lsr2 causes bridging of the linear DNA strands (Fig. S2*B*), as reported previously (60).

### HbhA deletion has no impact on *in vitro* growth of *Mtb*

**Figure 4:**
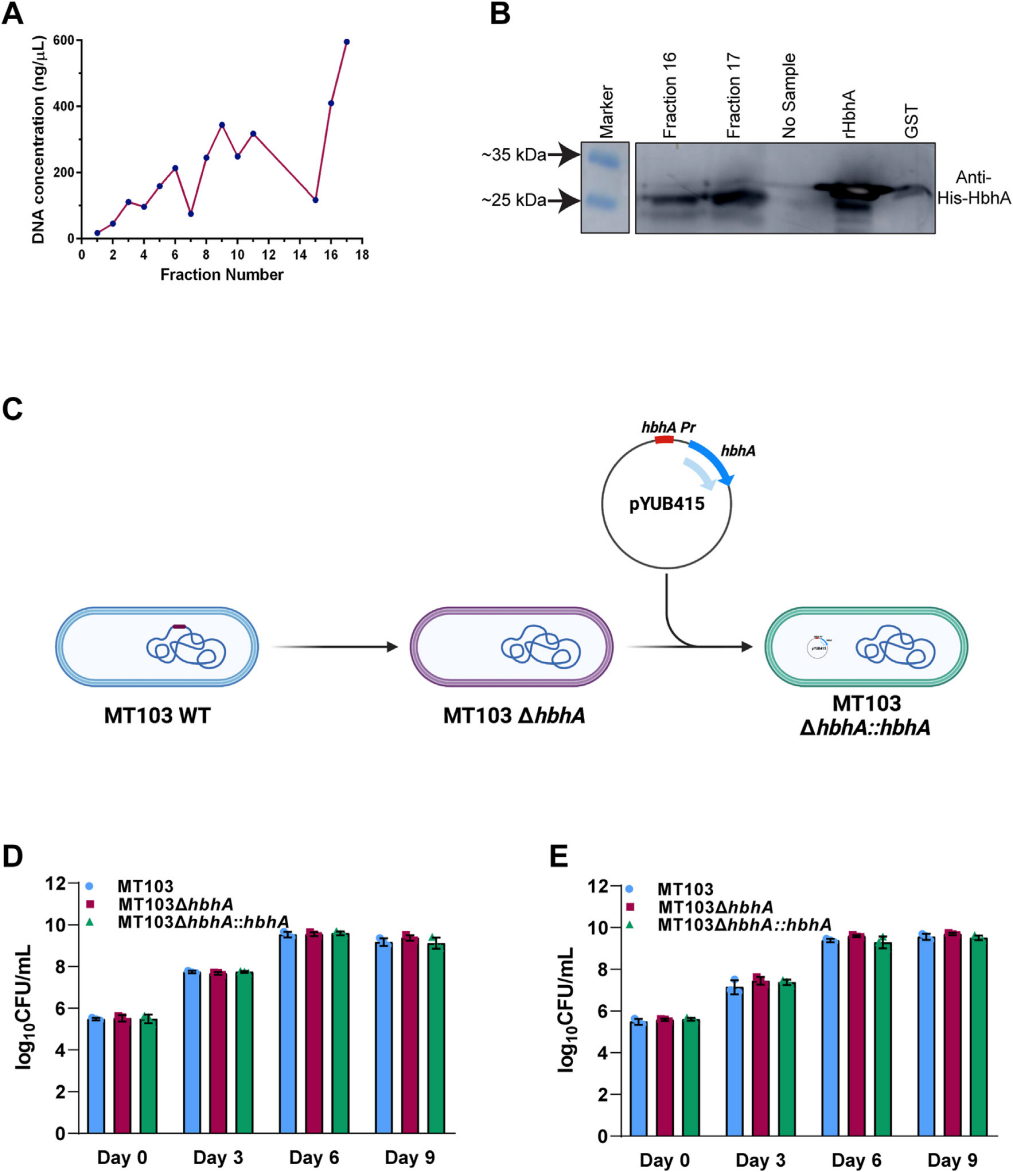
**HbhA colocalizes with nucleoid of *Mtb* and is nonessential for *invitro* growth of *Mtb*.***A*, nucleoids were isolated using 12 to 60% sucrose density gradient centrifugation. DNA concentration (ng/μl) in each fractions of 500 μl were plotted against fraction number in a XY-graph. Fractions with highest DNA content represent the nucleoid fractions. *B*, representative immunoblot of three independent experiments showing the presence of HbhA protein in nucleoid fractions. rHbhA represents His-HbhA recombinant purified protein. The presence of HbhA was detected using antibodies generated in mice against His-HbhA at 1:5000. *C*, a schematic representation of MT103, MT103Δ*hbhA*, and MT103Δ*hbhA::hbhA* strains. *D* and *E*, stationary phase cultures of MT103, MT103Δ*hbhA*, and MT103Δ*hbhA::hbhA* strains were inoculated at an initial *A*_600_ of 0.05 in 7H9 (*D*) and Sauton's (*E*) medium. Growth kinetics were measured by enumerating CFUs at indicated time points. Data represented shows mean log_10_ CFU/ml ± SD of independent biological triplicates. CFU, colony-forming units; HbhA, heparin-binding hemagglutinin; *Mtb*, *Mycobacterium tuberculosis*.

**Figure S3:**
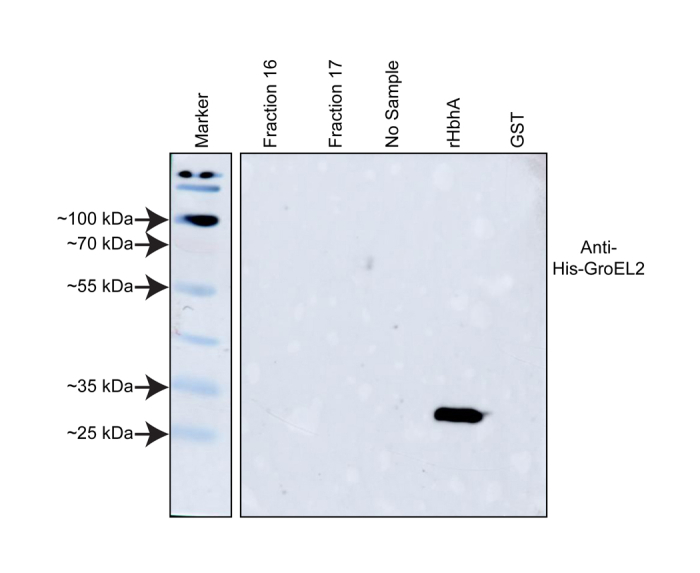
**Evaluation of the presence of GroEL2 with nucleoids of *Mtb*.** Immunoblot showing the absence of GroEL2 protein in nucleoid fractions of *Mtb*. rHbhA represents His-HbhA recombinant purified protein. The presence of GroEL2 was detected using antibodies generated in mice against His-GroEL2 at 1:10,000.

Next, we determined whether HbhA colocalizes with the nucleoid. *Mtb* nucleoids were isolated using sucrose density gradient centrifugation, and the concentration of DNA was measured in the various fractions (Fig. 4*A*). Fractions containing the highest amounts of DNA (Fractions 16 and 17) were considered nucleoid fractions (Fig. 4*A*) (54). Colocalization of HbhA with nucleoids was examined by immunoblot analyses, and the fractions with the highest amount of DNA were probed with anti-HbhA antibodies for the presence of HbhA. The presence of HbhA in fractions 16 and 17 indicates the colocalization of HbhA with nucleoids (Fig. 4*B*). To determine the specificity of HbhA-nucleoid colocalization, the presence of a cytosolic protein GroEL2 was also examined and was found absent in the nucleoid fractions (Fig. S3).

To examine the role of HbhA on the growth of *Mtb*, we compared the growth kinetics of WT *Mtb*103 (*MT103*) with those of the *hbhA* deleted strain *MT103ΔhbhA* and the complemented strain *MT103ΔhbhA::hbhA* (Fig. 4*C*) (43) in nutrient-rich 7H9 medium and nutrient-limiting Sauton's medium. There were no significant differences in the growth patterns of all three strains (Fig. 4, *D* and *E*), indicating that HbhA has no impact on the *in vitro* survival of *Mtb*.

### HbhA acts as a global transcriptional regulator

**Figure 5:**
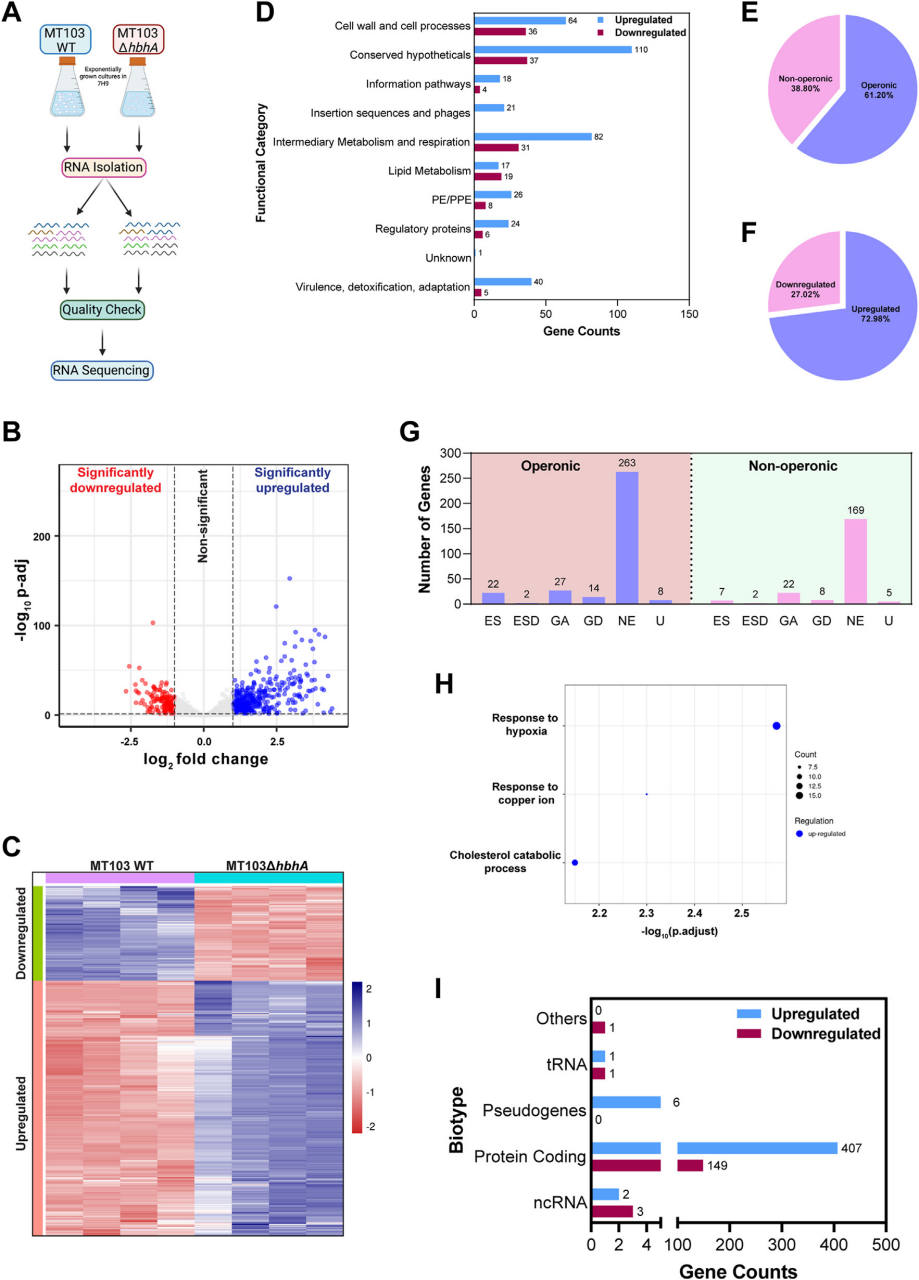
**HbhA acts as a global transcriptional regulator.***A*, schematic representation of RNA-sequencing experiment. *B*, volcano plot showing the differentially expressed genes (DEGs) in *MT103ΔhbhA* in comparison to *MT103*. *Red* and *blue* dots denote significantly downregulated and upregulated genes upon applying a cutoff of log_2_ fold change > 1.0 and adjusted *p*-value < 0.05. *C*, heatmaps showing the normalized read counts of all DEGs from four biological replicates of *MT103* and *MT103ΔhbhA*. *Red* and *blue* color intensities indicate relative downregulation and upregulation, respectively. *D*, bar graph showing the upregulated (*blue*) and downregulated (*red*) DEGs belonging to various functional categories. *E*, the pie chart depicting percentage of DEGs that are operonic and nonoperonic. *F*, the pie chart depicting the percentage of DEGs that are significantly upregulated and downregulated. *G*, bar graph depicting the number of operonic and nonoperonic DEGs belonging to essential (ES), essential domain (ESD), growth advantage (GA), growth defect (GD), nonessential (NE), and uncertain (U) categories. *H*, bubble plot depicting significantly enriched biological process categories of upregulated DEGs. *I*, bar graph depicting the number of significantly upregulated (*blue*) and downregulated (*red*) DEGs belonging to different biotypes. DEGs, differentially expressed genes; HbhA, heparin-binding hemagglutinin.

Since NAPs also act as global transcriptional regulators (6), we investigated the possibility of HbhA being a transcriptional regulator. To define the HbhA regulon, the transcriptomes of *MT103* and *MT103ΔhbhA* strains were compared by RNA-sequence analyses of four independent biological replicates (Fig. 5*A*). Differentially expressed genes (DEGs) were identified using DESeq2 analysis and represented as the volcano plot, where blue and red dots indicate significantly upregulated and downregulated genes, respectively [log_2_ fold change (log_2_fc) >1.0 and adjusted *p*-value (padj) < 0.05] (Fig. 5*B* and Table S1, *A* and *B*). A complete set of significantly upregulated and downregulated genes for all four biological replicates of *MT103* and *MT103ΔhbhA* are shown in the heatmap (Fig. 5*C*). Pathway analysis suggests that DEGs belong to multiple pathways such as virulence, detoxification, adaptation, cell wall synthesis and cell processes, lipid metabolism, and intermediary metabolism and respiration (Fig. 5*D*). Operonic arrangement analysis of DEGs showed that 38.80% of total DEGs are non-operonic, and 61.20% are operonic genes (Fig. 5*E*). HbhA deletion resulted in differential expression of a total of 570 genes (log_2_fc > 1.0 and padj < 0.05), of which 416 genes (72.98% of total DEGs) were upregulated, and 154 genes (27.02% of total DEGs) were downregulated (Fig. 5*F*). These results indicate that HbhA is a plausible transcriptional repressor. Of the 570 DEGs, 29 genes (22 operonic and seven non-operonic) were annotated as essential (Fig. 5*G*). Gene enrichment analysis showed that critical virulence and metabolic pathways such as response to hypoxia, response to copper ion, and cholesterol catabolic processes are significantly enriched in upregulated genes upon deletion of HbhA (Fig. 5*H* and Table S2, *A* and *B*). Biotype analysis showed that ∼98% of all DEGs are protein-coding genes (Fig. 5*I*). By applying a cutoff of log_2_fc>0.5 and padj<0.05, we found that expression of ∼36% of the total *Mtb* genes (1444 genes) and ∼29% of all essential genes are regulated by HbhA (Table S3, *A* and *B*). These results show that HbhA is a novel NAP, although deletion has no impact on *in-vitro* survival.

## Discussion

Bacteria can survive through various stress conditions. To cope with the stress, they have acquired numerous group- or species-specific and universal adaptive mechanisms (61, 62, 63). Among these mechanisms, modulation in the topology of the entire chromosome or within the specific regions of the chromosome is a ubiquitous, efficient, and effective strategy for adaptation (64, 65, 66). Chromosomal topology within bacterial cells is maintained by several factors, including macromolecular crowding, depletion forces, DNA supercoiling, and NAPs (5, 6, 67, 68, 69, 70). Using conserved motifs (Fig. 1*A*) present in histone proteins, we searched for the presence of those motifs in the proteome of *Mtb* and found four proteins (HupB, HbhA, RplV, and Rv3852) (Fig. 1*B*) out of which two (HupB and Rv3852) (39, 40) are already well-characterized NAPs. The remaining two proteins RplV and HbhA were purified and their DNA binding ability was tested. RplV failed to bind to DNA in a gel shift assay (Fig. 1*E*). It is known to specifically bind to 23S rRNA (https://mycobrowser.epfl.ch/genes/Rv0706). On the other hand, HbhA showed efficient interaction with DNA. Therefore, further experiments were performed to understand the DNA-binding activities of HbhA and its role in the life cycle of *Mtb* as a NAP.

NAPs act as chromatin-architectural proteins because of their ability to interact with DNA at multiple places in a nonspecific manner (5). Although NAPs can bind to DNA in a sequence- and structure-independent manner, some of them prefer particular DNA sequences or structures. For example, H-NS from *E. coli* and *Salmonella* (71, 72), HupB, Lsr2, NapM, and NapA from *Mtb* (21, 24, 36, 41) show preferential binding to AT-rich DNA segments and downregulate the expression of horizontally acquired genes that are AT-rich (73). Similarly, several NAPs have a preferential binding affinity for different structures or forms of DNA. For example, CbpA from *E. coli* shows preferential binding for curved DNA (74), Lsr2 and HupB from *Mtb* show preferential binding to supercoiled DNA and cruciform DNA (Holliday junctions), respectively (29, 75). We observed highly efficient binding of HbhA to both supercoiled and linear forms of pUC19 DNA (Fig. 2, *A* and *B*), which led us to conclude that HbhA is a promiscuous DNA-binding protein.

A closer look at the primary structure of HbhA revealed the presence of a lysine-, alanine-, and proline-rich region in its carboxy-terminal domain, with five AKKA repeats (Fig. 1*C*). The C-terminal domain of HbhA was previously reported to associate with sulfated glycoconjugates present on the host epithelial cell membrane (76). Several other studies have linked the presence of lysine-, proline-, and alanine-rich repeats in histone H1/H5 with their DNA-binding ability (37, 38, 52). The HU homolog in *Mtb* possesses a highly basic extension containing multiple repeats of lysine-, alanine-, and proline-rich repeats in their C terminal end. The C-terminal region of HupB containing AKKA/PAKKA repeats is also responsible for the efficient binding of HupB to DNA (24). Our data indicate that the C-terminal domain harboring similar motifs is necessary and sufficient for the DNA-binding activity of HbhA (Fig. 2*D*).

Histones and other histone-like proteins or NAPs are known to protect DNA from damaging agents such as DNaseI (53) or hydroxyl radical cleavage (28), and act as the transcriptional regulators (56, 57, 77). HU homologs encoded by *Deinococcus radiodurans* protect the genome from several damaging agents like ionizing and UV radiations, and help survive the bacterium in extreme environmental conditions (78). Further, Lsr2, GroEL1, HupB, mIHF, NapM, and NapA from *Mtb* also protect DNA from DNaseI digestion and hydroxyl radical cleavage (21, 24, 28, 29, 36, 54, 55). We show that HbhA also protects DNA from DNaseI-mediated degradation (Fig. 3*A*). Lsr2, a functional homolog of H-NS in *Mtb*, acts as a repressor of the horizontally acquired genes (41) in addition to regulating the expression of genes involved in physiology, antibiotic tolerance, and latency (27, 29). HU homologs are involved in the transcriptional regulation of genes involved in metabolism, general physiology, and virulence in case of *Salmonella* and *Francisella tularensis* (79, 80). The mycobacterial HU homolog, HupB regulates *katG* gene expression (26, 81), whose product is involved in activating isoniazid. Other mycobacterial NAPs such as mIHF, EspR, NapM, and NapA also regulate the expression of genes associated with general physiology, metabolism, and virulence (14, 21, 33, 36). We observed that HbhA acts as a transcriptional regulator since its binding inhibits transcription in an *in vitro* transcription assay (Fig. 3*B*).

NAPs protect DNA from damaging agents and act as global transcriptional regulators by modulating DNA topological and structural features. DNA supercoiling plays a major role in regulating gene expression and in maintaining the chromosomal architecture (82, 83). The introduction of negative supercoils in the DNA results in the opening of promoter regions and makes them accessible for RNA polymerase (84, 85). Further, NAPs such as Lsr2, HupB, and NapA regulate virulence-associated genes by inducing topological modulations in DNA in response to the environmental cues (21, 29, 75, 86, 87). We have observed that HbhA enhances topoisomerase I-mediated relaxation of DNA (Fig. 3*C*). Atomic force microscopic visualization of HbhA-DNA complexes revealed that HbhA also acts as a chromatin architectural protein since it introduces bends and loops in DNA (Fig. 3, *D* and *E*). NAPs are considered prokaryotic histone counterparts, as these proteins can induce structural changes in DNA by binding with it through various modes (bridging, bending, and wrapping) (5). For example, H-NS and its homologs from various bacterial species regulate gene expression and modulate bacterial chromosome architecture by bridging and coating the DNA (60, 88, 89).

The biochemical features of HbhA indicated that it could be a NAP. We also checked the localization of HbhA with the nucleoid of *Mtb* by isolating the nucleoids and detecting the presence of HbhA. We showed that HbhA is indeed associated with the nucleoid region of *Mtb* (Fig. 4, *A* and *B*), consistent with our hypothesis that HbhA is a NAP. Previous studies showed different localization patterns of HbhA. It was found to be present on the mycobacterial cell surface, secreted outside the mycobacterial cells and localized to the host cell mitochondria, and also present within the mycobacterial cells (90, 91, 92). HupB, a NAP of *Mtb*, and its homologs in *Mycobacterium smegmatis* and *Mycobacterium leprae* are also present on mycobacterial cell surface (93, 94, 95). HU, a highly conserved NAP throughout the prokaryotes, is detected on the bacterial cell surface and secreted outside the bacterial cell (96). In addition to prokaryotic NAPs, eukaryotic histones are also secreted in the extracellular milieu (97). However, the regulatory mechanisms behind multiple spatial localization of these proteins have not been elucidated.

**Figure S4:**
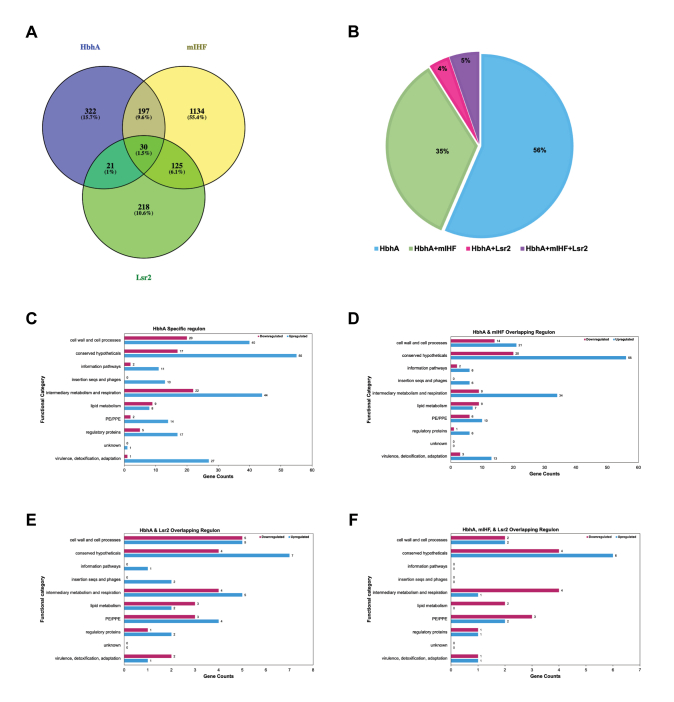
**Comparative analyses of HbhA, mIHF, and Lsr2 regulons.***A*, venn-diagram showing the percentage of overlap and uniqueness of HbhA, mIHF, and Lsr2 regulons upon applying a cut-off of log_2_ fold change > 1.0 and adjusted p-value < 0.05. *B*, the pie-chart showing the percentage of HbhA regulon that overlaps with mIHF, Lsr2, both mIHF and Lsr2, and unique to HbhA. *C*–*F*, bar graph showing the upregulated (*blue*) and downregulated (*red*) DEGs of (C) HbhA specific regulon, (*D*) HbhA and mIHF overlapping regulon, (E) HbhA and Lsr2 overlapping regulon, and (*F*) HbhA, mIHF, and Lsr2 overlapping regulon belonging to various functional categories.

HbhA deletion had no impact on the growth of *Mtb* in nutrient-rich 7H9 and nutrient-limiting Sauton's media (Fig. 4, *D* and *E*), suggesting that HbhA is a nonessential gene for *in vitro* survival of *Mtb* as reported earlier (32, 98, 99). While HbhA is nonessential for the *in vivo* survival of *Mtb*, it is suggested to be involved in extrapulmonary dissemination because of its adhesion property with sulfated glycoconjugates present on the host epithelial cell membrane through its C-terminal domain (43). NAPs also tend to act as global transcriptional regulator (6). The HbhA regulon was examined by comparing the global transcriptional changes in the *hbhA* deleted strain to its parental counterpart. The transcriptomic study showed differential expression of ∼36% of the total *Mtb* genes and ∼29% of all essential genes (log_2_fc > 0.5 and padj < 0.05) (Table S3). These DEGs belong to multiple critical metabolic and virulence pathways such as virulence, detoxification, adaptation, cell wall synthesis and cell processes, lipid metabolism, and intermediary metabolism and respiration (Fig. 5*D*). ∼73% of all DEGs showed upregulated expression upon *hbhA* deletion (Fig. 5*E*), suggesting HbhA as a plausible transcriptional repressor. Other *Mtb* NAPs, such as Lsr2 and mIHF, also regulate the expression of genes globally (27, 33, 41). Comparative analyses of the regulons of Lsr2 (27), mIHF (33), and HbhA showed that 35% and 4% of the HbhA regulon overlapped with the regulons of mIHF and Lsr2, respectively, and 5% of the HbhA regulon was shared by both mIHF and Lsr2 (Fig. S4, *A* and *B* and Table S4, *B*–*D*). This shows that HbhA is functionally similar to mIHF. The majority of DEGs of these NAPs belong mainly to cell wall and cell processes and intermediary metabolism and respiration (Fig. S4, *C*–*F* and Table S4, *A*–*D*).

In summary, we have characterized HbhA as a novel nucleoid-associated protein in *Mtb*, based on NAPs-like biochemical features such as DNA protection, transcriptional regulation, and topological and architectural modulations in DNA. The C-terminal domain of HbhA is the DNA-binding domain. Our results also showed that HbhA acts as a global transcriptional regulator. Even though deletion of *hbhA* influences the transcription of many essential genes, it is nonessential for *in vitro* growth in a complete medium. The transcriptional modulation observed of the essential genes may not be sufficient to display the eventual phenotypic impact on growth. The subsequent study can investigate the specific physiological relevance of the DNA-binding activity of HbhA.

## Experimental procedures

### Animal experimentation

All the animal experiment protocols were reviewed and approved by the Institutional Animal Ethics Committee of Department of Zoology, University of Delhi, India (the approved protocol number is DU/ZOOL/IAECR/2019). Balb/c mice were used for raising polyclonal antibodies used in the manuscript in accordance with the guidelines issued by the Committee for the Purpose of Control and Supervision of Experiments on Animals, Govt. of India.

### Bacterial strains and growth conditions

*E. coli* DH5α was used for cloning of the *Mtb* genes. For the expression of recombinant proteins, *E. coli* BL21 (Rossetta) was used as a surrogate host. *E. coli* cells were grown in LB broth at 37 °C, 200 rpm, and on LB agar medium at 37 °C (static condition). Ampicillin and chloramphenicol were added at a final concentration of 100 μg/ml, and 34 μg/ml, respectively, wherever required. *Mtb* strains were grown in 7H9 Broth (Difco) supplemented with 0.85 g/L NaCl, 5 g/L BSA, 2 g/L dextrose, and 4 mg/L catalase (ADC), 0.2% glycerol, 0.05% Tween 80 at 37 °C, 200 rpm and on 7H10 Agar (Difco) supplemented with 0.85 g/L NaCl, 5 g/L BSA, 2 g/L dextrose, 4 mg/L catalase, and 50 mg/L oleic acid, 0.5% glycerol at 37 °C. Proper aeration (1:5 head space ratio) was maintained during the bacterial growth. Bacterial strains used or generated during this study are listed in Table 1.

**Table 1:** List of bacterial strains used in this study

Name	Genotype	Resistance Marker	Reference
DH5α	*E. coli* F^-^Φ80*lac*ZΔM15 Δ(*lac*ZYA-*arg*F) U169 *rec*A1 *end*A1 *hsd*R17(r_k_^-^, m_k_^+^) *pho*A *sup*E44 *thi*-1 *gyr*A96 *rel*A1 λ^-^	-	Invitrogen
DH5α-pETPhos	*E. coli* DH5α strain producing pETPhos plasmid vector	Ampicillin	This study
DH5α-pETPhos-*rplV*	*E. coli* DH5α strain producing pETPhos-*rplV* plasmid	Ampicillin	This study
DH5α-pETPhos-*hbhA*	*E. coli* DH5α strain producing pETPhos-*hbhA* plasmid	Ampicillin	This study
DH5α-pETPhos-*lsr2*	*E. coli* DH5α strain producing pETPhos-*lsr2* plasmid	Ampicillin	This study
DH5α-pETPhos-*rv0475*_*1-160*_	*E. coli* DH5α strain producing pETPhos-*rv0475*_*1-160*_ plasmid	Ampicillin	This Study
DH5α-pProExHTc	*E. coli* DH5α strain producing pProExHTc plasmid vector	Ampicillin	This study
DH5α-pProExHTc-*rv0475*_*151-199*_	*E. coli* DH5α strain producing pProExHTc-*rv0475*_*151-199*_ plasmid	Ampicillin	This study
DH5α-pProExHTc- *rv0475*_*161-199*_	*E. coli* DH5α strain producing pProExHTc- *rv0475*_*161-199*_ plasmid	Ampicillin	This study
DH5α-pGEX-5X3	*E. coli* DH5α strain producing pGEX-5X3 plasmid vector	Ampicillin	This study
DH5α-pUC19	*E. coli* DH5α strain producing pUC19 plasmid vector	Ampicillin	This study
Rossetta (DE3)	*E. coli* F^-^ *ompT hsdS*_B_(r_B_^-^ m_B_^-^) *gal dcm* (DE3) pRARE (Cam^R^)	Chloramphenicol	Merck
Rossetta (DE3)- pETPhos-*rplV*	*E. coli* Rossetta (DE3) strain having plamid pETPhos-*rplV*	Ampicillin and chloramphenicol	This study
Rossetta (DE3)- pETPhos-*hbhA*	*E. coli* Rossetta (DE3) strain having plamid pETPhos-*hbhA*	Ampicillin and chloramphenicol	This study
Rossetta (DE3)- pETPhos-*lsr2*	*E. coli* Rossetta (DE3) strain having plamid pETPhos-*lsr2*	Ampicillin and chloramphenicol	This study
Rossetta (DE3)- pETPhos-*rv0475*_*1-160*_	*E. coli* Rossetta (DE3) strain having plamid pETPhos-*rv0475*_*1-160*_	Ampicillin and chloramphenicol	This study
Rossetta (DE3)- pProExHTc-*rv0475*_*151-199*_	*E. coli* Rossetta (DE3) strain having plamid pProExHTc-*rv0475*_*151-199*_	Ampicillin and chloramphenicol	This study
Rossetta (DE3)- pProExHTc-*rv0475*_*161-199*_	*E. coli* Rossetta (DE3) strain having plamid pProExHTc-*rv0475*_*161-199*_	Ampicillin and chloramphenicol	This study
BL21(DE3)	*E. coli* B strain*: F*^*–*^*ompT gal dcm lon hsdS*_*B*_*(r*_*B*_^*–*^*m*_*B*_^*–*^*) λ(DE3 [lacI lacUV5-T7p07 ind1 sam7 nin5]) [malB*^*+*^*]*_*K-12*_*(λ*^*S*^*)*	-	Invitrogen
BL21(DE3)-pGEX-5X3	*E. coli* BL21 (DE3) strain having plamid pGEX-5X3	Ampicillin	This study
H37Rv	WT *Mycobacterium tuberculosis* H37Rv strain	-	ATCC
*MT103*	WT *Mycobacterium tuberculosis* 103 strain	-	(43)
*MT103ΔhbhA*	*hbhA* deleted mutant of *Mycobacterium tuberculosis* 103 strain	Kanamycin	(43)
*MT103ΔhbhA::hbhA*	*hbhA* complemented strain of MT103Δ*hbhA*	Kanamycin and hygromycin	(43)

ATCC, American Type Culture Collection.

### Cloning, expression, and purification of recombinant proteins

Genes *rplV*, *lsr2, hbhA* (*rv0475*), *hbhAΔC* (coding for the N-terminal fragment containing amino acids 1–160), *hbhA*_*151-199*_, and *hbhA*_*161-199*_ (Coding for *hbhA* C-terminal domains, residues 151–199 and 161–199, respectively) were PCR amplified using the *Mtb* H37Rv genomic DNA as the template and primer pairs listed in Table 2. The amplicons were digested with respective restriction endonucleases and ligated into the corresponding sites of the previously digested expression vector pETPhos (Table 3).

**Table 2:** List of primers used in this study

S.No.	Primers' name	Primers' sequence^a^ (5′-3′)
P1	RplV FP^b^	GAACATATGACTGCGGCTACTAAGGCTACCG (NdeI)
P2	RplV RP	CTTGGATCCCTAGTCTGAGCCTCCCTTCGCTGCAG (BamHI)
P3	HbhA FP	GTTCATATGGCTGAAAACTCGAACATTGATG (NdeI)
P4	HbhA RP	GATGGATCCCTACTTCTGGGTGACCTTCTTGGCC (BamHI)
P5	Lsr2 FP	GAACATATGGCGAAGAAAGTAACCGTCACC (NdeI)
P6	Lsr2 RP	GATGCTAGCTCAGGTCGCCGCGTGGTATGCGTCG (NheI)
P7	HbhAΔC RP	GGAGGATCCTACTTAGGCAGCTCGATGCCGACC (BamHI)
P8	HbhA_151-199_ FP	CGCGGATCCGTGCCGCCAAGCTGGTCGGCATCGAGC (BamHI)
P9	HbhA_161-199_ FP	GCCGCCAAGCTGGTCGGATCCCTAAGAAGGCTG (BamHI)
P10	HbhA_CTD_ RP Common	GAGCCTCGAGCTACTTCTGGGTGACCTTCTTGGC (XhoI)

a Restriction sites are underlined and specified in the parentheses after the sequence.

b FP, Forward primer; RP, Reverse primer.

**Table 3:** List of plasmids used in this study

Name	Description	Resistance marker	Reference
pETPhos	Plasmid used for recombinant protein expression in *E. coli* with N-terminal 6X-His tag	Ampicillin	Invitrogen
pETPhos-*rplV*	Expression of 6X-His-RplV in *E. coli*	Ampicillin	This study
pETPhos-*hbhA*	Expression of 6X-His-HbhA in *E. coli*	Ampicillin	This study
pETPhos-*lsr2*	Expression of 6X-His-Lsr2 in *E. coli*	Ampicillin	This study
pETPhos-*rv0475*_*1-160*_	Expression of 6X-His-HbhAΔC in *E. coli*	Ampicillin	This study
pProExHTc	Plasmid used for recombinant protein expression in *E. coli* with N-terminal 6X-His tag	Ampicillin	Invitrogen
pProExHTc-*rv0475*_*151-199*_	Expression of 6X-His-HbhA_151-199_ in *E. coli*	Ampicillin	This study
pProExHTc-*rv0475*_*161-199*_	Expression of 6X-His-HbhA_161-199_ in *E. coli*	Ampicillin	This study
pGEX-5X3	Plasmid used for recombinant protein expression in *E. coli* with N-terminal GST tag	Ampicillin	Invitrogen
pUC19	*E. coli* cloning vector	Ampicillin	Invitrogen
pGEM	Plasmid having T7 promoter used for *in-vitro* transcription assay	Ampicillin	Promega

For the expression of recombinant proteins, *E. coli* BL21 (Rossetta) containing the generated constructs were grown overnight and used as inoculum for 1 L cultures. Expression was induced at *A*_600_ ∼0.6 using 1 mM IPTG at 37 °C for 3 h. Cells were pelleted and washed with PBS and stored at −80 °C until further processed. 6X-His-tagged recombinant proteins were purified as described previously (51, 100). Briefly, cells thawed on ice and resuspended in sonication buffer (50 mM Tris–Cl (pH 8.5), 300 mM NaCl, 10% glycerol, 20 mM imidazole, 1 mg/ml lysozyme, 1 mM PMSF, 1X protease inhibitor cocktail and 5 mM β-mercaptoethanol) and incubated on ice for 1 h with mild shaking. Cells were sonicated for nine pulses comprising 10 s on and 30 s off cycle. After sonication, the lysate was centrifuged at 16,000 rpm for 20 min, and the supernatant containing soluble recombinant protein was collected and loaded onto a Ni-NTA Superflow resin column, previously equilibrated with equilibration buffer A (50 mM Tris–Cl (pH 8.5), 300 mM NaCl, and 10% glycerol), buffer B (buffer A having 20 mM imidazole) and finally with 5 to 10 ml of sonication buffer. The column was washed with wash buffer A (50 mM Tris–Cl (pH 8.5), 300 mM NaCl, 10% glycerol, 20 mM imidazole, 1 mM PMSF, and 5 mM β-mercaptoethanol), wash buffer B (50 mM Tris–Cl (pH 8.5), 1 M NaCl, 10% glycerol, 20 mM imidazole, 1 mM PMSF, and 5 mM β-mercaptoethanol), again wash buffer A, and wash buffer C (50 mM Tris–Cl (pH 8.5), 300 mM NaCl, 10% glycerol, 50 mM imidazole, 1 mM PMSF, and 5 mM β-mercaptoethanol). Recombinant proteins were eluted with 50 mM Tris–Cl (pH 8.5), 150 mM NaCl, 10% glycerol, 200 mM imidazole. GST protein was purified as described (100). The purity of recombinant proteins was evaluated on 12% SDS-PAGE gel.

### Electrophoretic mobility shift assay

DNA-protein interaction studies were performed using EMSAs as described previously (51), with slight modifications. Briefly, for studying the interaction of proteins with nonspecific DNA template, pUC19 was used as a probe. DNA mixed with proteins at various DNA::protein molar ratios (1:0, 1:100, 1:250, 1:500, and 1:1000) were incubated at 25 °C for 30 min in a reaction buffer (10 mM Tris–Cl (pH 8.0), 50 mM NaCl, 50 μg/ml bovine serum albumin (BSA), 1 mM DTT, 1 mM EDTA, and 5% glycerol) and resolved on 0.5% agarose gel using 1× Tris-borate-EDTA, running buffer. Ethidium bromide (EtBr) was used for visualization of native DNA and DNA-protein complexes.

### DNaseI protection assay

DNaseI protection assays were carried out as described (24) with minor modifications. Briefly, 500 ng of pUC19 DNA was incubated with HbhA at molar ratios of 1:0, 1:250, 1:500, and 1:1000 (DNA::Protein) in the presence of a reaction buffer (100 mM Tris–Cl (pH 7.5), 25 mM MgCl_2_, and 5 mM CaCl_2_) at 25 °C for 30 min. An amount of 1 U of DNaseI (Invitrogen) was added, and the reaction was carried out at 37 °C for 30 s. The reactions were terminated by adding 1% SDS and 5 mM EDTA (final concentration), followed by electrophoresis on 1% agarose gel in 1X Tris-acetate-EDTA buffer. EtBr staining was used to visualize the DNA bands on the gel.

### *In vitro* transcription assay

*In vitro* transcription assays were performed following the manufacturer’s instruction (Riboprobe *in vitro* transcription assay kit, Promega) with slight modifications. Briefly, 250 ng of pGEM DNA was incubated with the various proteins at a molar ratio of 1:500 (DNA::Protein) for HbhA and HbhAΔC in 1X transcription buffer at 25 °C for 30 min. A total of 10 U of T7 RNA polymerase was added in a final reaction volume of 10 μl and further incubated at 37 °C for 1 h. Reaction products were resolved using 1% agarose gel in 1X Tris-acetate-EDTA buffer and visualized by EtBr staining of the gel.

### Topoisomerase I dependent DNA relaxation assay

Effect of HbhA on the supercoiling of plasmid DNA was studied according to the earlier described protocol (101). Briefly, 750 ng of pUC19 DNA was relaxed using 1 U *E. coli* Topoisomerase IA (New England Biolabs) in the presence of 1X CutSmart Buffer in a total reaction volume of 20 μl at 37 °C for 1 h. Relaxed DNA was incubated with HbhA at a molar ratio of 1:1000 (DNA::Protein) at 25 °C for 30 min. Reactions were terminated by the addition of Proteinase K and incubation at 37 °C for 15 min. Samples were resolved in a 1% agarose gel in 1X Tris-borate-EDTA buffer and stained with EtBr for visualization.

### Atomic force microscopy

Supercoiled or linear forms of pUC19 DNA were incubated for 30 min at 25 °C with each of the proteins (HbhA, HbhAΔC, and Lsr2) at a molar ratio of 1:250. Ten microliters of the reaction mixture was loaded onto a freshly cleaved mica surface (Ted Pella, Inc). The unbound material was washed with filtered MilliQ water, and the mica was air dried. Images were acquired by using a 5500 scanning probe microscope (Keysight Technologies, Inc) and Keysight Technologies PicoView 1.20.3 software (https://www.keysight.com/de/de/lib/software-detail/instrument-firmware-software/picoview-software/picoview-software--version-1-20-3.html#) in tapping mode in air with 125-μm-long silicon cantilevers (AppNano) with a resonance frequency of 200 to 400 kHz and a force constant of 13 to 77 N/m. The scan speed used was 1 line/s. Minimum image processing (first-order flattening and brightness contrast) was employed. Image analysis was performed using Pico Image Elements software v7.3 (https://www.keysight.com/in/en/assets/7018-01697/data-sheets/5989-7596.pdf).

### Colocalization of HbhA with nucleoid of *Mtb*

To investigate the colocalization of HbhA with nucleoids of *Mtb*; nucleoids were isolated by sucrose-density gradient centrifugation as described (54). Briefly, glycine was added at a final concentration of 1% to exponentially growing cultures and incubated for 2 h at 37 °C and 200 rpm. Cells were harvested, washed with PBS (pH 7.4), resuspended in 5 ml chilled acetone and incubated at −20 °C for 1 h. Cells were harvested, air dried, resuspended in 1 ml extraction buffer containing 20% sucrose, 1 mg/ml lysozyme, and 1X Protease Inhibitor cocktail (Roche), and incubated overnight at 37 °C and 200 rpm. Finally, 1% of NP-40 was added to the mixture, incubated for 2 h at 37 °C and 200 rpm, and centrifuged at 3000*g* to remove the debris. Lysates containing nucleoids were passed through a 0.22 μm filter. Cleared lysates were layered onto a 12 to 60% sucrose density gradient and was centrifuged at 4000*g* and 4 °C for 15 min in a swing bucket rotor. The tube's bottom was punctured to collect sucrose gradient fractions of 500 μl each. The presence of DNA in the fractions was measured at *A*_260 nm_, and the amount of DNA was plotted against the fraction numbers. The fractions containing the highest amount of DNA were considered nucleoid-containing fractions.

The *Mtb* nucleoid fractions were resolved using a 12% SDS-PAGE and transferred onto the nitrocellulose membrane. The presence of HbhA with nucleoid region was detected using antibodies by immunoblotting.

### Immunoblotting

For immunoblotting, nucleoid fractions containing 40 μg proteins were separated on 12% SDS-PAGE and transferred to nitrocellulose membranes (Millipore). After blocking with 3% BSA in PBS containing 0.05% Tween 20 (PBST) for 1 h, membranes were washed three times with PBST and incubated overnight with primary antibodies anti-His-HbhA (1:5000) or anti-His-GroEL2 (1:10,000) antibodies at 4 °C. These antibodies were generated in mice using purified recombinant proteins as antigens. One percent of BSA was added to prevent nonspecific antibody binding. Membranes were washed five times with PBST and treated with anti-mouse immunoglobulin G antibody conjugated to horseradish peroxidase (1:10,000-Cell Signaling Technology, Cat. No. 7076S) for an hour. Membranes were washed again with PBST five times and finally developed using SuperSignal West Pico PLUS/Femto chemiluminescent substrate (Thermo Fisher Scientific).

### Growth kinetics, RNA isolation, and transcriptomics

Exponentially grown *Mtb* cultures (*MT103*, *MT103ΔhbhA*, and *MT103ΔhbhA::hbhA*) in the 7H9-ADC medium were inoculated at an initial *A*_600 nm_ of 0.05 in nutrient-rich 7H9 or nutrient-limited Sauton's media and allowed to grow at 37 °C, 200 rpm. Growth kinetics was monitored by measuring *A*_600_ every 24 h, and serial dilutions of cultures were plated on 7H11 agar plates for colony-forming units enumeration.

RNA was isolated using TRIzol reagent (Ambion). Cell numbers equivalent to *A*_600_ of 10 of exponentially grown *MT103* and *MT103ΔhbhA* strains were harvested by centrifugation and resuspended in TRIzol reagent. Following cell lysis using 0.1 mm zirconium beads, RNA was extracted with chloroform, precipitated with isopropanol, and washed with 75% ethanol. RNA pellets were air dried and dissolved in nuclease free water (102). Isolated RNA was subjected to DNaseI treatment to remove DNA contamination from the samples.

RNA isolated from four independent biological replicates were used for RNA-sequencing and transcriptomics analyses as per the earlier described protocol (102). Sequencing was performed at the core sequencing facility of the Centre for Cellular and Molecular Biology, Hyderabad, India.

### Functional enrichment analysis

DAVID web services (https://david.ncifcrf.gov) were used for functional enrichment analysis. Only GO terms and KEGG pathways were selected for gene enrichment analysis.

### Statistical analysis

Data reproducibility was ensured by performing experiments in three independent biological replicates, unless stated otherwise. Data plotting and statistical significance calculation were performed using GraphPad Prism software (Prism 9) (https://www.graphpad.com/features). Two-way ANOVA followed by a post hoc Tukey's test was employed to analyze statistical significance.

### Figures preparation

Bio Render was used to prepare illustrations/schematics, whereas ImageJ and Adobe Illustrator were used to prepare scientific figures.

## Data availability

RNA-seq data are available at the NCBI Gene Expression Omnibus Database with accession number GSE235293 (https://www.ncbi.nlm.nih.gov/geo/query/acc.cgi?acc=GSE235293).

## Supporting information

This article contains supporting information.

## Conflict of interest

The authors declare that they have no conflicts of interest with the contents of this article.
