# Supplementary material for: The heparin-binding hemagglutinin protein of Mycobacterium tuberculosis is a nucleoid-associated protein
Source: J Biol Chem. 2023 Oct 19;299(12):105364. doi: 10.1016/j.jbc.2023.105364 (PMC10665949; doi:10.1016/j.jbc.2023.105364)
Supplement: Supporting information [file mmc1.docx]

**SUPPORTING INFORMATION**

**The heparin-binding haemagglutinin protein of *Mycobacterium tuberculosis* is a nucleoid-associated protein**

Chetkar Chandra Keshavam, Saba Naz, Aanchal Gupta, Priyadarshini Sanyal, Manisha Kochar, Aakriti Gangwal, Nitika Sangwan, Nishant Kumar, Ekta Tyagi, Simran Goel, Nitesh Kumar Singh, Divya Tej Sowpati, Garima Khare, Munia Ganguli, Dominique Raze, Camille Locht, Sharmila Basu-Modak, Meetu Gupta, Vinay Kumar Nandicoori, Yogendra Singh

List of supplementary information included:

**Figure S1.** Multiple sequence alignment (MSA) and phylogenetic analysis of HbhA from different actinobacterial species.

**Figure S2.** Effect of HbhAΔC and Lsr2 on the architectural features of linear pUC19 DNA.

**Figure S3.** Evaluation of the presence of GroEL2 with nucleoids of *Mtb*.

**Figure S4.** Comparative analyses of HbhA, mIHF, and Lsr2 regulons.

**Table S1.** DEGs analysis of MT103 vs MT103Δ*hbhA* (log2fc>1.0 & padj<0.05). a) Upregulated genes b) Downregulated genes.

**Table S2.** Functional enrichment analysis upon the deletion of HbhA. a) Downregulated b) Upregulated genes.

**Table S3.** DEG analysis of MT103 vs MT103*ΔhbhA* (log2fc>0.5 & padj<0.05). a) Downregulated b) Upregulated genes.

**Table S4.** Comparative analyses of HbhA, mIHF, and Lsr2 regulons. a) HbhA specific regulon, b) HbhA and mIHF overlapping regulon, C) HbhA and Lsr2 overlapping regulon, and d) HbhA, mIHF, and Lsr2 overlapping regulon.
